# UNDERSTANDING THE DYNAMICS OF INTRINSIC HEALTH USING AGE-DEPENDENT QUANTILE GRAPHICAL MODELS

**DOI:** 10.1093/geroni/igad104.0118

**Published:** 2023-12-21

**Authors:** Zain Khan, Daniel Malinsky, Alan Cohen, Sen Pei, Kiros Berhane, Martin Picard, Daniel Belsky, Ying Wei

**Affiliations:** Columbia University, New York City, New York, United States; Columbia University, New York City, New York, United States; Columbia University, New York City, New York, United States; Columbia University, New York City, New York, United States; Columbia University, New York City, New York, United States; Columbia University Irving Medical Center, New York City, New York, United States; Columbia University, New York City, New York, United States; Columbia University, New York City, New York, United States

## Abstract

Human health is a complex and dynamic system. Individual components, such as clinical biomarkers, vary jointly as an ensemble, and appropriate models are needed to study them in the context of health. Network analysis is an effective way to assess and visualize cross-biomarker dependencies, where each node represents a biomarker, and each edge represents the conditional dependency between two biomarkers. We developed a new class of age-dependent quantile graphical models to fully capture the complexity of cross-biomarker dynamics. This model quantifies cross-biomarker dependencies at the boundary (i.e., when biomarkers approach or exceed normal ranges) and differentiates them from those within normal ranges. Further incorporation of age-dependency helps identify biological mechanisms by which humans maintain intrinsic health over their life course. Applying this analysis to the NHANES data (n=60,014), we discovered that cross-biomarker dependencies differed by age and across quantile levels. For participants under 20 years old, at the median quantile we found significant relationships between heart rate (HR) and all other biomarkers: cholesterol, hemoglobin, albumin, systolic blood pressure (SBP), and diastolic blood pressure (DBP) (all p-values < 0.001). However, elderly populations (60+) at the median quantile displayed cholesterol centrality instead—albumin, SBP, DBP, and HR were significantly associated with cholesterol (all p-values < 0.001) while HR was less connected to other biomarkers. At the 0.9 quantile, the <20 group resembled the older age groups in that cholesterol was the more central node. This demonstrates that association levels and centrality patterns varied across age groups and quantile levels.

